# Advances in synthetic peptide immuno-regulatory epitopes

**DOI:** 10.1186/1939-4551-7-30

**Published:** 2014-11-10

**Authors:** Peter Socrates Creticos

**Affiliations:** 1 Creticos Research Group, Crownsville, Maryland USA; 2 Division of Allergy & Clinical Immunology, Johns Hopkins University School of Medicine, Baltimore, Maryland USA

**Keywords:** Allergen Immunotherapy (AIT), Subcutaneous Injection Immunotherapy (SCIT), Late-phase Skin Responses (LPSR), Nmol Group, Nmol Dose

## Abstract

Synthetic peptide immuno-regulatory epitopes (SPIRE) represent a new class of therapeutics for allergen immunotherapy that offer the potential to suppress the IgE-mediated allergic disease process through induction of T-cell tolerance. These synthetic T-cell-tolerizing peptides have been designed to induce immunologic tolerance via binding to MHC class II molecules on antigen presenting cells, with subsequent upregulation of regulatory T-cells.

**Electronic supplementary material:**

The online version of this article (doi:10.1186/1939-4551-7-30) contains supplementary material, which is available to authorized users.

## Introduction

Specific allergen immunotherapy (AIT) is employed to attenuate or tolerize the immune response to specific allergens and is recommended for patients in whom allergic rhinitis symptoms cannot be controlled by medication and environmental control, or in patients that cannot tolerate their medications, or in patients that are non-compliant with chronic medication regimens [1, 2].

AIT has been shown to reduce the immediate phase of clinical reactivity (mast cell mediator release), inhibit cellular recruitment (eosinophils; basophils) in the late phase, and resolve the underlying inflammatory process that characterizes allergic rhinitis. The mechanisms by which this is accomplished continue to be investigated, but are currently thought to include a reduction of allergen-specific Th2 T-cell responses mediated by increased Th1 or regulatory T cell responses to the same allergen [3, 4].

Conventional subcutaneous (SCIT) injection regimens employ increasing doses of the allergen extract, administered once or twice weekly, as tolerated, until the patient reaches a predetermined “maintenance” dose (e.g. 6–12 μg Amb a 1 (2000–4000 BAU)/injection). The optimal duration of SCIT with a licensed allergen extract is not well defined; but, general recommendations are to administer the maintenance dose one or twice monthly over 3–5 years [1, 2]. However, this approach is not optimal, as it is limited by the potential for systemic allergic reactions, including anaphylaxis, it utilizes a tedious long-term treatment regimen that negatively impacts upon patient compliance, and in some patients it is only partially effective. Hence, there is a need for newer therapeutic agents that can be administered more easily (either fewer injections or a different route of immunization), are safer (have a reduced risk of serious adverse reactions), and can provide effective benefit to a larger percentage of patients.

Through the years, various chemical modifications of allergen have been attempted to enhance efficacy, improve safety, and foster compliance with AIT. Recent approaches with modified allergens, adjuvants, including immune-stimulatory adjuvants, recombinant allergens, T-cell tolerizing constructs, and improved oral approaches have been demonstrated in various clinical studies to provide measurable benefit in treatment of allergic respiratory disease [5–9].

### Synthetic T-cell-tolerizing peptides

Studies carried out by Gefter and colleagues, and other investigators, in the mid-1990s provided the first evidence that synthetic T-cell-tolerizing peptides could induce tolerance and thereby be used to suppress IgE-mediated diseases such as cat and ragweed-induced rhinitis and asthma. His lab developed both cat (two 27 amino acid peptides derived from *Fel d 1*) and ragweed T-cell-tolerizing peptides and in collaborative studies with Norman, Creticos et al. demonstrated that various treatment regimens, in which the peptides were administered subcutaneously, provided significant improvement in cat-induced clinical symptoms in the cat studies (cat room challenges; cat broncho-provocation studies), and of allergic rhinoconjunctivitis symptoms in multicenter trials of a ragweed vaccine. Furthermore, the peptides did not induce an increase in antibody response – suggesting immune tolerance [10–15].

However, these first-generation peptides were longer sequences, fewer in number, and given subcutaneously at much higher doses; in clinical trials, they suffered from a late-onset adverse event profile, and a therapeutic result that was less than optimal when contrasted to conventional SCIT. Development of this approach was halted in the late-1990s.

New promising research into peptide epitopes initiated at Imperial College by Larché and Kay has resulted in a second generation of these molecules with the subsequent development of Synthetic Peptide Immuno-Regulatory Epitopes (SPIREs). These synthetic T-cell-tolerizing peptides are comprised of smaller peptide units (e.g.: cat: seven peptides; 13–17 amino acids in length), administered in much smaller quantities (75 μg vs. 750 μg), are assembled from different T-cell epitopes and are administered intradermally to access antigen presenting cells [16–22].

These novel peptides are designed to induce immunologic tolerance via binding to MHC class II molecules on antigen presenting cells, with subsequent up-regulation of regulatory T-cells. A key advantage of the peptides lies in their smaller size; i.e., molecules that are of insufficient length to trigger cross-linking of IgE on mast cells and basophils, may provide an advantage by potentially reducing the risk of IgE-mediated allergic reactions such as asthma, urticaria, or anaphylaxis [16–22].

### Cat-SPIRE

#### Cat-SPIRE development

Cat-SPIRE [Circassia Ltd.; Oxford, U.K.] consists of 7 separate small peptides (13–17 AA in length) derived from *Fel d 1*, the major cat allergenic moiety. It is prepared as a lyophilisate^a^ and must first be reconstituted in sterile water for intradermal injection into the dermis [21, 23] (^a^ see Endnotes).

The selection of peptides in Cat SPIRE were pre-defined based on MHC class II binding studies using appropriate cell lines and upon conducting T-cell proliferation and histamine release assays on ex-vivo blood samples from cat allergic human volunteers in order to identify the specific T-cell epitopes of interest. T-cell proliferative responses to cat dander allergen extract and Cat-SPIRE correlated closely and provided confirmatory evidence that the majority of T cell reactivity to cat dander could be ascribed to the specific epitopes contained within Cat-SPIRE. As importantly, the cytokine responses against whole cat dander and Cat-SPIRE showed a skewing toward a T-regulatory IL-10 protective response [19, 21, 23].

Furthermore, as important as the identification and selection of the appropriate T-cell epitopes for Cat-SPIRE, the assessment and determination of the compound’s IgE-binding activity was equally critical to the design of the therapeutic agent. Basophil histamine release assays on whole blood from cat-allergic individuals affirmed that Cat-SPIRE possessed significantly less potential than whole allergen to cross-link IgE thereby reducing the potential to cause either local injection reactions or systemic reactions [16–23].

A synthetic mixture of T cell epitopes (Cat SPIRE) capable of being administered in a short 4-injection treatment regimen, with a more practical intradermal method of application (as opposed to a subcutaneous injection), affords a therapeutic construct that offers a significant improvement to the method of administration of immunotherapy - one that has the potential to positively impact upon patient compliance. The animal and human investigational studies to date have provided promising evidence to support that long-lasting clinical benefit can be achieved with this approach [24].

### Animal studies

An initial mechanistic study by Campbell et al. in transgenic mice demonstrated that treatment with a specific cat peptide (one of the peptides in Cat-SPIRE) resulted in reductions in BAL (bronchoalveolar lavage) total cells and eosinophils (cells indicative of allergic inflammation), reductions in pulmonary and systemic TH2 inflammatory cytokines, reduced recruitment of TH2 cells to the lungs, and reduced proliferative responses to *Fel d 1*[19].

Administration of an anti-IL-10 monoclonal antibody immediately after treatment with the peptide blocked these positive peptide-induced effects and provided immunologic evidence that IL-10 is a critical cytokine in the underlying mechanism of action ascribed to SPIREs for re-establishment of immune tolerance.

Although this is an artificial acute sensitization animal model, its findings are consistent with the current understanding of the role of regulatory T-cells in allergen immunotherapy. Furthermore, the parallel observations that mixtures of peptides from Cat-SPIRE induced *in-vitro* IL-10 release from peripheral blood mononuclear cells (PBMCs) from >90% of cat-allergic subjects provides additional evidence for the ability of the peptide construct to induce clinical tolerance [19].

### Clinical studies

#### Phase I/IIa safety and efficacy study

Worm et al. published their initial safety and efficacy findings on *Fel d 1* peptide immunotherapy in 2011 [21]. Cat-SPIRE was administered in a double-blind, placebo-controlled clinical trial which employed an escalating single-dose intradermal (ID) or subcutaneous (SQ) injections to evaluate safety and efficacy in 88 cat allergic subjects [21].

The primary endpoint was safety and tolerability, and the primary surrogate efficacy endpoint was defined as the change in mean diameter of the LPSR (late phase skin response) vs. placebo at 8-hours post-ID challenge with whole cat allergen on Day 21 after receipt of Cat-SPIRE or placebo. That is a well-recognized clinical endpoint to assess the effect of immunotherapy in allergic subjects. It was determined that the greatest inhibition of the LPSR response to ID whole allergen challenge was observed with the 3 nmol dose (~37.5 mcg of *Fel d 1* peptides). This dose resulted in approximately a 40% reduction in the LPSR vs. 10% for placebo. This change did not achieve statistical significance, which given the small number of subjects involved is not unexpected. However, the positive trend combined with the antibody data helped to define the subsequent treatment dose for future development [21].

Cat-SPIRE was well-tolerated in doses up to 12 nmol (given ID) and 20 nmol (given SQ), as well as no clinically significant changes in any laboratory parameter, abnormalities on ECG testing, or adverse findings on physical exam were observed. No serious adverse events (SAEs) were observed during the study, and no subject withdrew from the study due to an adverse event. The most common treatment-emergent adverse events in the ID-immunized subjects were nasopharyngitis, cough, and headache; whereas, in the SQ-immunized subjects, nasal congestion and respiratory symptoms were reported - not unlike what might be observed with SCIT (subcutaneous injection immunotherapy) to whole cat extract [21].

Furthermore, no subjects in the ID-immunized cohort exhibited a reduction in FEV_1_ greater that 20% from baseline (considered indicative of a meaningful asthmatic response) and none reported any asthma-like symptoms during the 8-hour post-dosing period [21, 25].

In comparison to SCIT, it is recognized that the effective maintenance dose for immunotherapy is ~15 mcg of *Fel d 1*, but that a buildup phase of approximately 4–6 months is required to achieve this dose level. Hence, the findings that Cat-SPIRE was safe and well-tolerated at a single dose of up to 12 nmol (ID) (equivalent to ~150 mcg of Fel d 1) provided initial confirmatory evidence that Cat-SPIRE could be administered at an effective dose that that might not necessitate a lengthy dose escalation or buildup phase [1, 2, 21].

#### Phase II clinical study

The Environmental Exposure Chamber (EEC) provides a controlled setting in which to evaluate drugs (e.g., antihistamines; nasal corticosteroids) and allergen immunotherapy. It provides a surrogate to field trials and avoids many of the challenges associated with outdoor seasonal (and perennial) immunotherapy studies (i.e., weather changes; pollutant exposures; confounding aeroallergen exposure) that can affect phase II multi-center studies. Furthermore, it is possible to expose patients to pre-defined allergen levels that are “expected” to cause symptoms of sufficient severity to allow assessment of drug effect [26].

This model was chosen to perform dose-ranging studies and to explore the dose-dependent clinical efficacy that might be expected with Cat-SPIRE immunotherapy in cat-allergic volunteers. In the EEC design, study subjects had a baseline EEC visit consisting of 4 consecutive days on which patients were exposed for three hours each day in the chamber. The subjects were then re-exposed in the EEC at 18–22 weeks after the start of treatment.

One-hundred and twenty-one (121) subjects who met a qualifying threshold symptom score were randomized to one of four treatment arms or placebo: a) 4 administrations of 3 nmol 2 weeks apart; b) 4 administrations of 6 nmol 2 weeks apart; c) 4 administrations of 3 nmol 4 weeks apart; d) 8 administrations of 3 nmol 2 weeks apart; e) 8 administrations of placebo; all dosing regimens included placebo infill injections where necessary to maintain the blind [27, 28].

The primary endpoint (defined as the difference in TRSS at each time point on each day between baseline and post-treatment challenge (PTC) demonstrated a greater degree of efficacy when the study subjects were dosed over 12–14 weeks as opposed to 6 weeks. Although not based on intent to treat (ITT) analysis (due to technical issues) those patients who attended the main study center and who received the 8 administrations of 3 nmol 2 weeks apart dose regimen reported a reduction of symptoms versus placebo (p < .05). A trend was also observed for the 6 nmol dose to be superior to the 3 nmol dose [27, 28].

No serious adverse events were observed in this study with any of the four treatment regimens. TEAEs in all cohorts (except the 6 nmol cohort) were less than the placebo cohort (the 6 nmol cohort trended slightly higher). However, respiratory system TEAEs (including asthma, dyspnea, and wheezing) occurred at a low frequency in all groups, and no difference was observed between active groups or placebo [27, 28].

Recognizing that the goal of immunotherapy is to induce a long-lasting treatment effect that is maintained post-cessation of treatment, this EEC study provided preliminary evidence that a sustained treatment effect could be achieved with Cat-SPIRE. Obviously, the 18–22 week findings were intriguing and led to additional EEC work to establish duration of treatment effect – that is the ability of a short course of immunotherapy with Cat-SPIRE to induce a long-lasting disease-modifying effect. The work by Durham et al. with SCIT has demonstrated that a 3-year course of immunotherapy with a modified standardized grass extract can induce sustained benefit in the 2-years post-cessation of treatment. This serves as the “benchmark” for new immunotherapy constructs [29].

#### Phase IIb clinical study with 1-year follow-up

The focus of this study, by Patel and colleagues, was to further define the persistence of treatment effect in study patients with cat-induced AR undergoing treatment with Cat-SPIRE [24].

This randomized, double-blind, placebo-controlled study used the same baseline challenge methodology as the earlier study (baseline challenge consisting of 4 consecutive days of 3 hours in the EEC) and had patients undergo cat challenge in the EEC at 18–22 weeks and at 50–54 weeks after the start of treatment. The primary endpoint was defined as the change in TRSS, (post-treatment vs. baseline EEC challenges) at 1–3 hours on days 2–4. The Cat-SPIRE regimen randomized 202 patients to either: a) 4 doses of 6-nmol 4 weeks apart (n = 66); b) 8 administrations of 3 nmol 2 weeks apart (n = 67); or c) placebo (n = 69) [24].

The results of this 1-year study demonstrated a persistent treatment effect in the pre-specified statistical analysis at time points after 1 hour on Days 2–4 of EEC at the 50–54 week EEC challenge in the non-asthmatic population for the 6 nmol × 4 dose regimen vs. placebo (median change: -6.80 (vs. -3.27); mean change: -3.89 +/-5.56 (vs. -2.91 +/-5.56); LS means: -7.074, -4.077; 95% CIs: -7.165 to -0.989; p value =0.0104). Analysis of the data at all time points on all days showed similar results [24].

Furthermore, the clinical effects were similar when assessing “all patients” (including patients with asthma) with the 6 nmol × 4 dose regimen vs. placebo (median change: -6.03 (vs -3.20); mean change: -6.35 +/-5.75 (vs. -2.49 +/-5.39); LS means: -6.507, -3.878; 95% CIs: -6.586 to -1.158; p value =0.0057). This finding demonstrates that asthmatic subjects not only tolerate the immunization regimen but likewise demonstrate a clinical benefit that is sustained and similar to that seen in the cohort with AR without asthma at the 1-year follow-up EEC challenge [24].

Analyses on the secondary endpoints of nasal and ocular symptoms, respectively, showed that similar benefit was seen in both of these parameters at the 1-year time point (total nasal symptom score (mean change): -3.44 +/- 3.05 (vs. -1.63 +/-2.95); p value vs. placebo =0.02 // total ocular symptom score (mean change): -3.34 +/- 3.05 (vs. -1.28 +/- 2.92); p value vs. placebo =0.01) and highlights the prominence of ocular symptoms in cat-induced AR [24].

**Figure 1:**
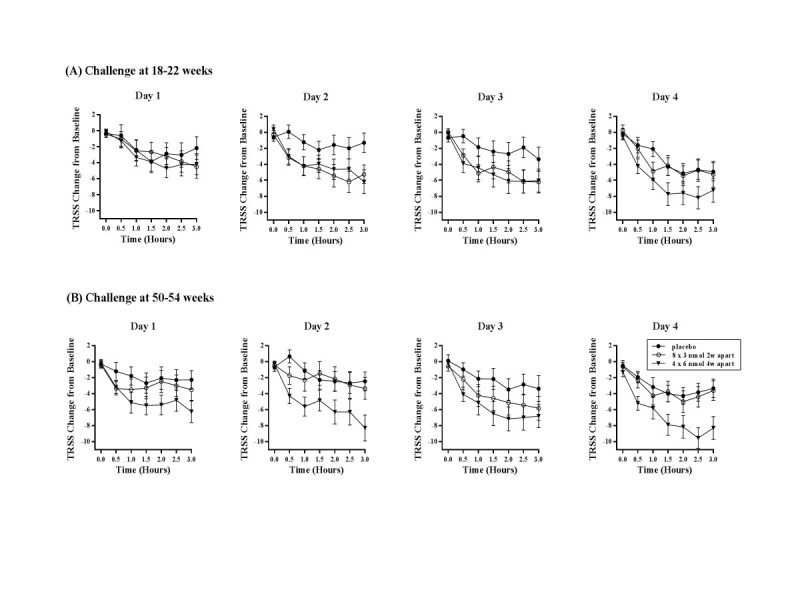
**Delta changes in total rhinoconjunctivitis symptom scores for the treatment effect observed with Cat-SPIRE in the environmental exposure chamber.**

Figure 1 displays the mean TRSS symptom scores at each 30-minute time point in the chamber over the 4 consecutive exposure days for the baseline EEC challenge and the 1-year EEC challenge, and in fact points out that the challenge performed at 1-year demonstrates the treatment effect to be stronger on successive days in the EEC [24].

The magnitude of change in TRSS scores for CAT-SPIRE (~4 TRSS units) compares favorably with allergen chamber studies of a SLIT cat allergy drops (change: 1.6u), or an antihistamine (change: 1.3u) in similar allergen EEC studies [23, 24].

Cat-SPIRE was reasonably well-tolerated. There was one serious AE (skin laceration) in a subject in the placebo group. There were no SAEs in the asthmatic subset of patients as related to their asthma. The majority of TEAEs were mild in severity, and no TEAEs were rated as severe. Six subjects did not complete the study because of a TEAE (1 on placebo (back pain); 5 in Cat-SPIRE groups: arthralgia and extremity pain (1 patient; 3 nmol); bronchospasm (2 patients that received 3 nmol); hypersensitivity (1 patient; 6 nmol); presyncope and convulsion (1 patient; 6 nmol)). Of note, save the hypersensitivity reaction (allergy symptoms self-medicated with an antihistamine and a *cold medicine*), the other 5 withdrawals due to TEAEs were assessed as unrelated to study drug [24].

Analysis of respiratory system-related TEAES did not elicit any untoward safety signal. There were no reductions in FEV1 of greater than 30%, (which was the prospectively defined cutoff, as based on clinical experience which provides a comfort level with bronchodilator responsiveness). Three subjects that received 6 nmol experienced an episode of dyspnea, bronchospasm, or asthma; whereas, 14 subjects that received 3 nmol and 11 subjects that received placebo reported such an episode [24].

The findings from this study extend the observations made in the earlier EEC clinical trial and provide evidence that a consistent and long-lasting effect on symptoms can be induced through immunization with Cat-SPIRE in cat-allergic individuals. The well-defined EEC model has proven to be a valuable tool in defining dose, determining treatment effect, and establishing clinical evidence for sustained therapeutic efficacy that is consistent with finding from animal models and *in-vitro* assays in cat-allergic subjects treated with immune-modulating peptides.

#### Phase IIb clinical study - two-year follow-Up

Of the 86 patients that completed all visits at the 1-year follow-up, 51 agreed to re-consent, remain blinded, and enroll in the 2-year follow-up study. No further treatment was administered [25, 30]. Study subjects underwent repeat chamber challenge at 102–106 weeks. As before, both subjects and study staff remained blinded.

A sustained improvement in mean TRSS from baseline was observed for the 6 nmol group (-5.87) as compared to the 3 nmol group (-3.05) and the placebo group (-2.02) at the post-treatment challenge (102–106 weeks after the start of treatment) (LS mean treatment difference (for the 6 nmol) vs. placebo was -3.85 (95% CI: -8.83, 1.14; p = 0.13). Although this trend did not reach significance for the primary endpoint, a statistically significant difference in findings was observed for pre-specified secondary endpoint when the cumulative allergen challenge was greatest (Day 4, 3 hours) (p = .02). Furthermore, consistent reductions in nasal symptoms (2–3 units) were observed in the 6 nmol group compared to placebo at multiple time points during the 4-day chamber exposure (e.g. the 2–3 hour time point in the EEC on days 1–4 (p = .05) [25, 30].

The findings from this 2-year follow-up study provide a degree of evidence that points to a persistence of effect, albeit limited, even at 2 years post-completion of a short course of immunization to Cat-SPIRE is encouraging. This *persistence of effect* will need to be evaluated further in larger studies.

#### Current phase III clinical field study

Recruitment for this clinical trial is underway in North America and Europe. This clinical trial aims to provide meaningful clinical data in a “real world” setting – that is, cat allergic patients who live with cats in their home environment.

This is a randomized, double-blind, placebo-controlled clinical trial of adolescents and adults (ages: 12–65). It is properly powered to include study subjects stratified (based on asthma status and age) in a 1:1:1 ratio to active therapy with either: a) 4 × 6 nmol of Cat-SPIRE doses 4 weeks apart (followed by 4 injections of placebo 4 weeks apart); or b) 4 × 6 nmol of Cat-SPIRE doses 4 weeks apart (followed by a second course of Cat-SPIRE 4 weeks apart); or c) placebo (two courses of 4 doses 4 weeks apart) [31].

The primary endpoint for this multi-center clinical trial is the mean CS (Combined Score: TRSS + RMS (Rhinoconjunctivitis Medication Score) during Weeks 52–54 after randomization in the Cat-SPIRE group compared to the mean CS in the placebo-treated group. In addition, multiple secondary endpoints of clinical efficacy will be measured in the clinical field trial [31].

### Other synthetic peptide immuno-regulatory epitope constructs in development

Recent work with ragweed SPIRE [32] and house dust mite SPIRE [33] have also provided initial evidence that these constructs can be delivered safely and demonstrate effect in skin, conjunctival, and/or environmental chamber provocation models.

## Conclusion

Synthetic peptides comprised of T-cell epitopes, whose sequence is derived from known amino acid sequences of specific allergens, provide a therapeutic approach which can be utilized to our advantage to induce long-lasting clinical efficacy.

## Endnote

^a^Study medication manufacture: The peptides were synthesized by Bachem (Bubendorf, Switzerland), according to current Good Manufacturing Practice. The lyophilized Cat-PAD product was formulated, filled, and finished by Patheon (Monza, Italy) and the lyophilized placebo was manufactured by Aptuit (Glasgow, United Kingdom), also according to current Good Manufacturing Practice. The materials were tested at Patheon and Gen-Probe (Livingston, United Kingdom) and released in accordance with the European Union (Directive 2001/20/EC) and Canadian (Food and Drug Act, Section C.05.010) regulations after labeling and packaging at Aptuit (Bathgate, United Kingdom).

## Authors’ original submitted files for images

Below are the links to the authors’ original submitted files for images. Authors’ original file for figure 1
