# Supplementary figures and images for: Advances in synthetic peptide immuno-regulatory epitopes
Source: World Allergy Organ J. 2014 Nov 10;7(1):30. doi: 10.1186/1939-4551-7-30 (PMC4230367; doi:10.1186/1939-4551-7-30)

## Slide 1
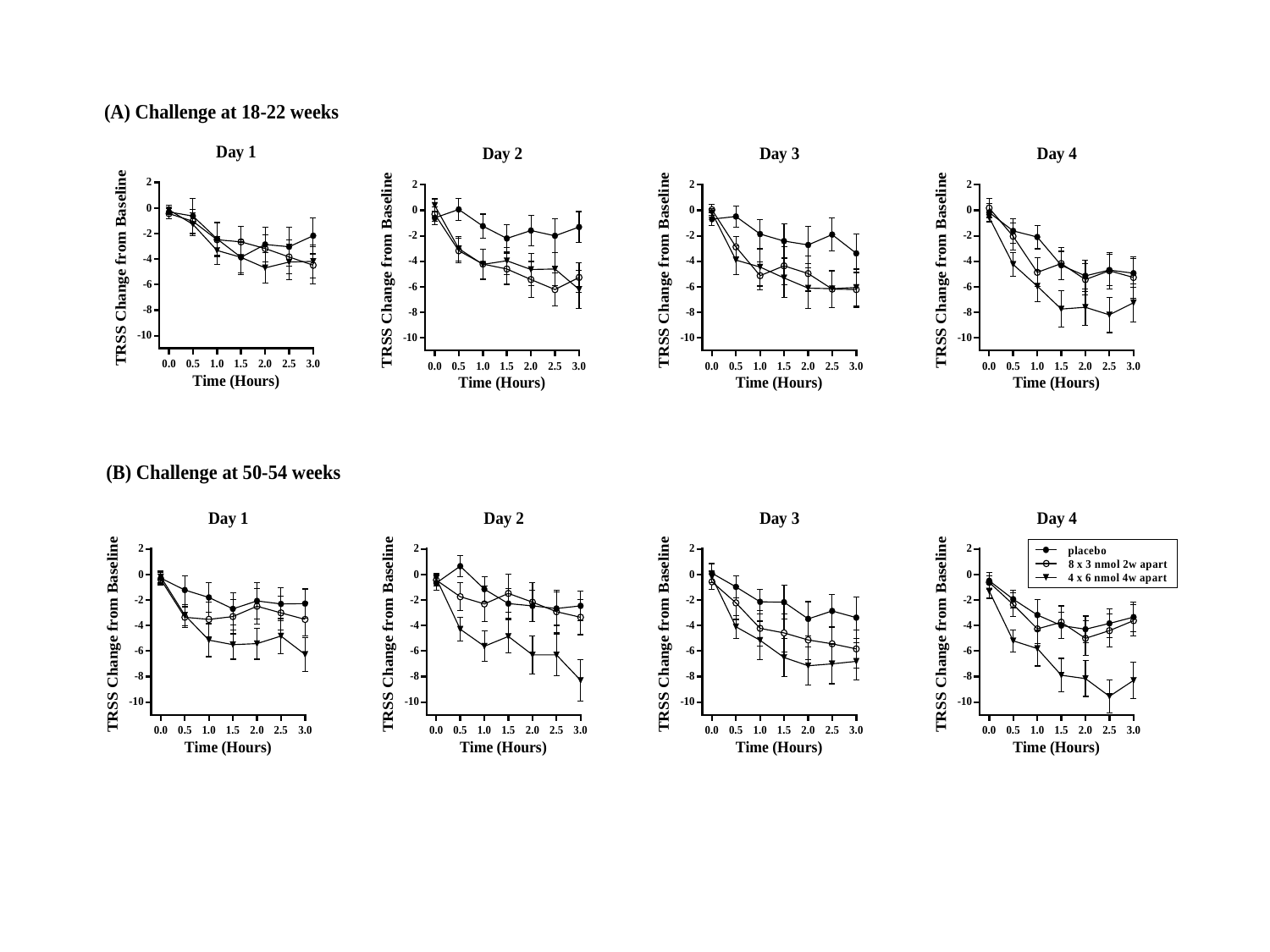

Supplement: Supplementary file 1 — Authors’ original file for figure 1 [file 40413_2014_71_MOESM1_ESM.ppt]
